# Health professionals’ competencies regarding breastfeeding beyond 12 months: a systematic review

**DOI:** 10.1186/s13006-023-00591-z

**Published:** 2023-10-31

**Authors:** Oona Ojantausta, Niina Pöyhönen, Riikka Ikonen, Marja Kaunonen

**Affiliations:** 1 Faculty of Social Sciences, Health Sciences, University of Tampere, Tampere, Finland; 2 Department of Nursing Science, Faculty of Health Sciences, University of Eastern Finland, Kuopio, Finland; 3 Pirkanmaa Wellbeing Services, General Administration, Tampere, Finland

**Keywords:** Breastfeeding, Lactation, Maternal-child health services, Health personnel, Clinical competence, Attitude of health personnel, Systematic review

## Abstract

**Background:**

Breastfeeding up to two years and beyond supports the health and neurobiological development of a child. Nevertheless, mothers experience criticism from health professionals towards breastfeeding beyond 12 months. Competence related to breastfeeding counselling is defined as minimum knowledge, skills and attitudes that all health professionals should have to protect, promote and support breastfeeding. Professionals’ education related to breastfeeding beyond 12 months is insufficient worldwide which challenges the competent and evidence-based support mothers wish for.

**Methods:**

This systematic review aimed to synthesize the existing literature on health professionals’ competencies regarding breastfeeding beyond 12 months. The search was limited to peer-reviewed scientific papers published between 2000 and 2022 that focused on the competencies of health professionals regarding breastfeeding beyond 12 months. Seven databases were searched, and of the 884 studies retrieved, seven were included in the review. The studies were subjected to the Joanna Briggs Institute (JBI) critical appraisal checklists. The data were analyzed using deductive thematic analysis, driven by the concept of competence.

**Results:**

All the dimensions of competence could be found in the data. Health professionals’ knowledge or skills related to breastfeeding beyond 12 months were explored in all seven studies, and attitudes towards breastfeeding beyond 12 months were explored in four studies. The main themes identified were Knowledge Combined with Skills, and Attitudes. The main theme, Knowledge Combined with Skills, was formed out of eight themes: perceptions regarding nutritional value, perceptions regarding economic value, perceptions regarding family interaction, perceptions regarding impacts on the mother’s wellbeing, perceptions regarding impacts on the child’s wellbeing, perceptions regarding suitable duration, perceptions regarding recommendations, and counseling skills. The attitudes varied and therefore the Attitudes main theme consisted of three themes: promotive attitudes, hostile attitudes, and passive attitudes towards breastfeeding beyond 12 months.

**Conclusions:**

Health professionals’ knowledge and skills include several dimensions and vary substantially. Health professionals’ attitudes vary between hostile and supportive and influence professionals’ advice regarding breastfeeding beyond 12 months. The results suggested that there is considerable variation in health professionals’ competencies, which emphasizes the importance of education regarding breastfeeding beyond 12 months.

**Supplementary Information:**

The online version contains supplementary material available at 10.1186/s13006-023-00591-z.

## Background

Breastfeeding up to two years and beyond supports the health and neurobiological development of an infant and a young child [1, 2]. A child aged over one year can get one-third of the recommended daily intake of calories and protein, and significant amounts of vitamins and minerals from breast milk [3]. However, mothers often face criticism and negative attitudes towards breastfeeding beyond 12 months [4–6] and feel pressure to stop breastfeeding at that point [5, 7].

In addition to negative attitudes in mothers’ social environment, support from health professionals may be insufficient. Health professionals may assume that the mother has stopped breastfeeding during the child’s first year of life and may not discuss breastfeeding with the mother after that milestone [4, 7]. Health professionals may even have negative attitudes and be critical towards breastfeeding beyond 12 months [5–8]. As a result, mothers have been left to feel that the approach towards breastfeeding depends on the individual health professional [7]. Moreover, the assumption of discontinuing breastfeeding or previously encountered negative attitudes have led mothers to avoid discussing their breastfeeding plans with health professionals [7–9]. A lack of trust has also been found to emerge as mothers have come to question health professionals’ competence [10] and instructions regarding breastfeeding beyond 12 months [4, 7, 10, 11]. Mothers have felt a lack of trust between themselves and health professionals because of the lack of knowledge and support [10] and this has led to mothers seeking support for breastfeeding primarily from peer support groups [4] to eliminate the fear of being judged [10]. Unsupportive responses from health professionals have also been a reason to switch healthcare providers [9].

Despite the WHO breastfeeding recommendations and the objectives of Baby Friendly Initiative [12], professional education related to breastfeeding beyond 12 months is insufficient worldwide [13]. In addition, health professionals’ own experiences affect their breastfeeding competencies [14], which may lead to unequal encounters with mothers as the professional’s personal experience can be either an incentive or a barrier in providing breastfeeding support [15]. There are no guidelines for counseling breastfeeding beyond 12 months and little is known about the customized counseling content for mothers who breastfeed longer. However, it is known that supporting breastfeeding beyond 12 months is different from supporting breastfeeding in the earlier months, as there are changes to the child’s nutritional needs, sleeping habits and family’s daily activities [8]. It is essential for health professionals to understand the meaning of breastfeeding beyond 12 months in the family’s everyday life and the fact that this can involve much more than just nutrition: mothers often use it as a parenting tool for soothing, calming, and putting the child to sleep [11]. This is often not understood in healthcare and mothers feel blamed for breastfeeding at night, and for using breastfeeding as a tool to calm down their child [4, 16].

This review explores health professionals’ competencies regarding breastfeeding beyond 12 months. Competence is a holistic term referring to a person’s ability to do something successfully [17] and achieve the desired result [18]. Health professionals’ competence can be based on interests and experiences influenced by motivation and attitudes [19], and it includes, in addition to the knowledge and skills required by the job, the individual's personality, attitudes and values [20]. In the context of nursing, clinical competence has been defined in several concept analyses [21–23] and studies [24, 25] as an entity consisting of knowledge, skills, and attitudes. In the context of breastfeeding, the United States Breastfeeding Committee (USBC) defines the breastfeeding core competencies as “*the minimal knowledge, skills, and attitudes necessary for health professionals from all disciplines to provide patient care that protects, promotes, and supports breastfeeding*” [26]. In this review, competencies refer to health professionals’ knowledge, skills, and attitudes regarding breastfeeding beyond 12 months. Health professionals refer to any health professionals or health students that interact with breastfeeding mothers at some point. This systematic review aims to synthesize the existing literature on health professionals’ competencies, namely knowledge, skills, and attitudes, regarding breastfeeding beyond 12 months.

## Methods

### Search strategy and screening

**Fig. 1:**
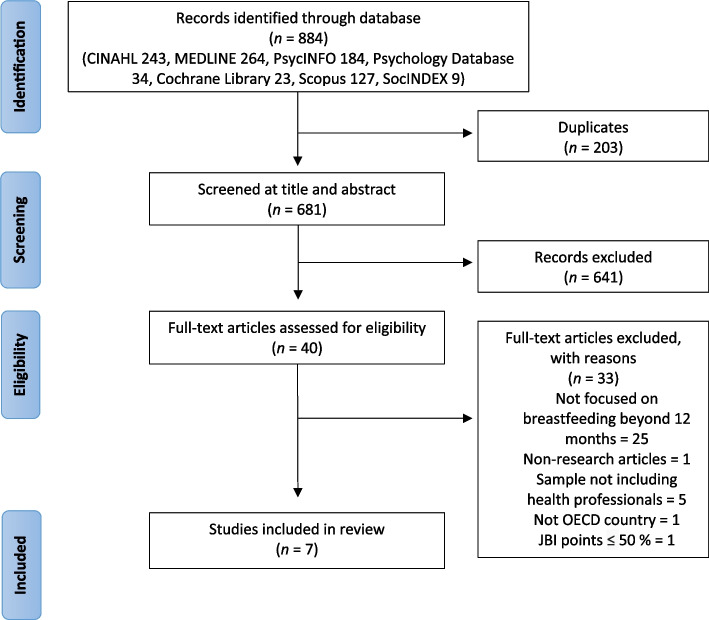
PRISMA flow chart of study selection

A literature search was carried out in December 2022. Seven databases (CINAHL, MEDLINE, PsycINFO, Psychology Database, Scopus, Cochrane Library and SocINDEX) were searched. The searches were limited to peer-reviewed scientific papers that were published between 2000 and December 2022. A relatively broad time limit was used to find a balance between the coverage of relevant studies on this little-studied phenomenon and a change in breastfeeding counseling culture over decades. Both medical subject headings (MeSH terms) and keywords were used. The searches were performed by combining related words and synonyms of “breastfeeding”, “prolonged” and “competence”. An additional file shows the searches in more detail (see Additional file 1). A total of 884 published articles were found and after excluding duplicates, 681 articles were included in the screening (Fig. 1).

### Study selection

The included studies focused on the competencies of health professionals regarding breastfeeding beyond 12 months. The studies were delimited to those published in The Organisation for Economic Co-operation and Development (OECD) countries because breastfeeding as a phenomenon is different and World Health Organization (WHO) breastfeeding recommendations are met differently in high-income countries than in developing countries [27]. All studies, regardless of their study design, that focused on exploring and reporting on health professionals’ competencies (namely knowledge, skills and/or attitudes) were considered eligible. Studies were assessed against pre-defined eligibility criteria.

#### Inclusion criteria

1. Original research article
2. Written in English
3. Studies focused on competencies of health professionals regarding breastfeeding beyond 12 months (namely knowledge, skills and/or attitudes)

#### Exclusion criteria

1. Studies not focused on breastfeeding beyond 12 months
2. Sample not including health professionals
3. Competencies not self-evaluated by healthcare professionals
4. Not an OECD country
5. Full text not available
6. JBI point ≤ 50%

The study selection was performed by four reviewers (OO, NP, MK, RI) who operated in pairs. In the first phase, the studies were divided into two parts and both reviewer pairs (OO & MK, NP & RI) read their own half of the abstracts. The eligibility criteria were used to eliminate 641 studies. All the studies that obviously or probably met the inclusion criteria and those that did not meet the exclusion criteria as clearly were taken to the next phase of study selection. The remaining 40 studies were selected for full-text screening. Both pairs read their half of the studies carefully. A total of 32 studies were excluded based on full-text screening: 25 studies did not concern breastfeeding beyond 12 months, one was not a research article, five studies had no health professionals in their samples, and one was from a non-OECD country. Any disagreements in the reviewer pairs were discussed in the group of four reviewers until a consensus was reached. After the study selection, a total of eight studies were accepted for quality assurance.

#### The quality of the studies

The included studies were subjected to the Joanna Briggs Institute (JBI) critical appraisal checklists by four researchers, who operated in pairs (OO & MK, NP & RI). Five of the studies [28–32] were assessed using the Joanna Briggs Institute Checklist for Analytical Cross-Sectional Studies. Three of the studies [33–35] were assessed using the Joanna Briggs Institute Checklist for Quasi-Experimental Studies. Studies had to score > 50% JBI points to be included in the review, and as a result, one study scoring 50% of the points was eliminated. At the end of the study selection and the quality appraising process, seven studies were included in the review.

### Data extraction and analysis

The purpose of this systematic review was to synthesize the existing literature on health professionals’ competencies (namely knowledge, skills, attitudes) regarding breastfeeding beyond 12 months. As thematic analysis has the ability to combine qualitative and quantitative findings and make qualitative synthesis of quantitative data [36], it was determined to be the most suitable analytical approach to meet the aim of the review. The data was analyzed using deductive thematic analysis [37], as it was driven by the definition of the concept of competence. Due to the heterogeneity in the quantitative study designs and outcomes, meta-analysis could not be performed.

The analysis was performed by two researchers (OO & NP) and the Atlas.ti data analysis software was used as a co-working platform in the process. At the beginning of the process, both researchers familiarized themselves carefully with all seven studies, making notes and highlighting quotations from the text. The quotations were chosen based on words or phrases that described an area of health professionals’ knowledge, skills, or attitudes related to breastfeeding beyond 12 months. After independent work, the researchers discussed their findings together and verified that there was a consensus on the 139 quotations found in the data. The researchers started the coding process together, discussing, and ensuring that all the codes would be consistent. To limit the effect of personal bias, both researchers coded the data and any difference in opinion was discussed. Theory-driven themes of different dimensions of competence directed the coding [37]. Forming the themes was carried out together by the same two researchers. A total of 101 codes were sorted to form preliminary themes. These themes were reviewed in relation to the codes and the original quotations from the data and organized again until there was certain that the original data was identifiable from the themes [37]. At the end of the process, there were eleven themes that were categorized into two main themes, Attitudes, and Knowledge combined with skills. Knowledge and skills were combined due to limited data. Knowledge and skills can also be considered to be partly overlapping because skills manifested through breastfeeding counseling that was directed by knowledge. An example of the progress of the analysis is provided in Table 1.

**Table 1:** An example of the progress of analysis

**Code **	**Code group **	**Theme **	**Main theme **
24 months of breastfeeding (bf) is considered “long breastfeeding” [30]	Having varying definition of extended bf [30]	Perceptions regarding appropriate bf duration [30–35]	Knowledge combined with skills
Defining extended bf as up to 12 months of bf [30]			
Negative perceptions increase along with the age of the child [33, 35]	Perceiving bf as less acceptable as the child gets older [33–35]		
Considering bf as more acceptable if younger toddler [33]			
Support for bf decreased after 6 months [34]			
Belief that children should be breastfed until over 24 months [30]	Perceiving bf beyond 24 months as desirable; [30, 33]		
Belief that it is acceptable for 3–4-year-old child to be breastfed [33]			
Encouraging to stop bf a 3- or 4-year-old child [32, 33]	Having varying perceptions about stopping bf [30–33]		
Perceiving bf beyond 12 months to complicate stopping bf [31]			
Believing that bf should be stopped before child turns 6 months [30]			
Stronger encouragement to stop bf when the child gets older [32]			

## Results

### Included studies

Seven studies were included in the review. The studies were published between 2012 and 2022 and had been conducted in the United States *n* = 3 [31–33], Italy *n* = 2 [29, 35], Poland *n* = 1 [30] and Canada *n* = 1 [34]. The smallest sample was *n* = 46 [34] and the largest was *n* = 4,582 [35]. The participants (*n* = 6,173) were physicians, pediatricians, gynecologists, neonatologists, midwives, public health nurses, nurses, pharmacists, medical residents, nursing students, and public health students. Table 2 includes a summary of the studies, including information about authors, publication year, location, aims, methodological approach, data collection, participants, relevant findings, themes identified from the study, and JBI scores.

**Table 2:** Characteristics of the included studies

**Author(s), year, country**	**Aims**	**Methodological approach, data collection, number of participants**	**Relevant findings**	**Themes identified from the study**	**JBI**
1. Baranowska [30] et al. (2019) Poland	To determine the level of knowledge and the attitudes of health professionals towards breastfeeding beyond 12 months; the relationship between personal breastfeeding experience and attitudes towards breastfeeding beyond 12 months; and the relationship between knowledge about breastfeeding beyond 12 months and attitudes towards it.	Cross-sectional study A one-group prospective, cross-sectional online survey *N* = 495, gynecologists, neonatologists, and midwives	Majority of the health professionals were not aware of the WHO recommendation of breastfeeding duration. Most of the health professionals had a low level of knowledge about the benefits of breastfeeding beyond 12 months. Health professionals thought breastfeeding beyond 12 months may indicate problems in the relationship between mother and child. Majority of the health professionals had negative or neutral attitudes towards breastfeeding beyond 12 months.	7/11 of all themes Perceptions regarding family interaction Perceptions regarding impacts on the child’s wellbeing Perceptions regarding appropriate breastfeeding duration Perceptions regarding the breastfeeding recommendations Attitudes promoting breastfeeding Hostile attitudes Passive attitudes	8/8^a^ Yes: 8 No: 0 Can’t tell: 0
2. Cockerham-Colas [33] et al. (2012) USA	To explore the knowledge and attitudes of health professionals towards breastfeeding beyond 12 months and to pilot an educational display for health professionals to promote their knowledge and attitudes towards breastfeeding beyond 12 months.	Quasi-Experimental study Pre and post surveys, structured questionnaire *N* = 84, health professionals (i.e., physicians, midwives, medical residents, nursing students, public health students etc.)	In general, health professionals had negative attitudes towards breastfeeding beyond 12 months. Health professionals thought breastfeeding was less acceptable as the child got older.	2/11 Perceptions regarding appropriate breastfeeding duration Hostile attitudes	7/9^b^ Yes: 7 No: 1 Can’t tell: 1
3. Colaceci [35] et al. (2020) Italy	To evaluate the long-term effectiveness of an online national program of infant nutrition for health professionals.	Quasi-Experimental study Data collection at three time points (T0, T1, T2) using questionnaires *N* = 4582, health professionals (i.e., nurses, midwives, physicians, pharmacists etc.)	Health professionals thought breastfeeding was less acceptable as the child got older.	2/11 Perceptions regarding appropriate breastfeeding duration Perceptions regarding the breastfeeding recommendations	7/9^b^ Yes: 7 No: 1 Can’t tell: 1
4. Radaelli [29] et al. (2012) Italy	To examine attitudes and practices of family pediatricians towards infant feeding.	Cross-sectional study Online questionnaire *N* = 850 pediatricians	Minority of pediatricians recommended breastfeeding beyond 12 months.	1/11 Perceptions regarding the breastfeeding recommendations	7/8^a^ Yes: 7 No: 1 Can’t tell: 0
5. Rempel, McCleary [34] (2012) Canada	To evaluate the effect of Breastfeeding Best Practice guideline implementation on health professionals’ knowledge, beliefs, and behavior regarding breastfeeding and breastfeeding promotion.	Quasi-Experimental study Pre and post surveys, structured questionnaires *N* = 46 public health nurses	Health professionals didn’t support the WHO recommendations of breastfeeding up to 2 years and beyond.	2/11 Perceptions regarding appropriate breastfeeding duration Perceptions regarding the breastfeeding recommendations	6/9^b^ Yes: 6 No: 3 Can’t tell: 0
6. Zhuang [31] et al. (2020) USA	To examine the perceived advantages and disadvantages, emotional responses, and advice that healthcare students would provide to mothers regarding breastfeeding beyond 12 months.	Cross-sectional study Online open-ended questionnaire *N* = 116 healthcare students	Majority of the health professionals could name some of the child’s health benefits of breastfeeding beyond 12 months. General benefits, as emotional and economical, and mother’s health benefits were not as widely known. Most of the health professionals named disadvantages of breastfeeding beyond 12 months. Health professionals had predominantly negative emotions and neutral responses to breastfeeding beyond 12 months.	10/11 Perceptions regarding nutritional value Perceptions regarding economic value Perceptions regarding family interaction Perceptions regarding impacts on the mother’s wellbeing Perceptions regarding impacts on the child’s wellbeing Perceptions regarding appropriate breastfeeding duration Counseling skills Attitudes promoting breastfeeding Hostile attitudes Passive attitudes	5/8^a^ Yes: 5 No: 1 Can’t tell: 2
7. Goldbort, Hitt, Zhuang [32] (2022) USA	To examine how extended breast- feeding is perceived among medical and nursing students and how perceptions of extended breast- feeding are translated into stigmatizing outcomes including attitudes, behavioral predispositions, and behavioral intention to encourage to stop breastfeeding.	Cross-sectional study Online close-ended qustionnaire *N* = 116 healthcare students	Health professionals had a lack of knowledge regarding breastfeeding beyond 12 months and increasingly negative attitudes as the child’s age increased. The intention to encourage to stop breastfeeding increased as the child got older.	4/11 Perceptions regarding appropriate breastfeeding duration Counseling skills Attitudes promoting breastfeeding Hostile attitudes	7/8^a^ Yes: 7 No: 0 Can’t tell: 1

^a^Checklist for Analytical Cross-Sectional Studies

^b^Checklist for Quasi-Experimental Studies

### Main theme 1: Knowledge Combined with Skills

All seven studies considered the health professionals’ knowledge or skills related to breastfeeding beyond 12 months [29–35]. The main theme, Knowledge Combined with Skills, was formed out of eight themes: perceptions regarding nutritional value [31], perceptions regarding economic value [31] perceptions regarding family interaction [30, 31], perceptions regarding impacts on the mother’s wellbeing [31], perceptions regarding impacts on the child’s wellbeing [30, 31], perceptions regarding suitable duration [30–35], perceptions regarding recommendations [29, 30, 32, 34, 35], and counseling skills [31, 32].

The perceptions regarding nutritional value varied. Health professionals found breastfeeding beyond 12 months nutritionally beneficial, which was linked to the knowledge of the nutritional benefits of breastfeeding and the view that breastfeeding provides good nutrition [31]. On the other hand, some health professionals perceived breastfeeding beyond 12 months as a risk for the child's nutrition. Health professionals recommended solids and considered breastfeeding to cause nutritional deficiency for the child [31].

Perceptions regarding economic value were explored in one study [31]. Health professionals acknowledged breastfeeding beyond 12 months as economically beneficial, and more economical than bottle feeding.

The perceptions regarding family interaction varied from considering breastfeeding beyond 12 months emotionally valuable [31] to finding it harmful to family relationships [30]. The view of breastfeeding as emotionally valuable was linked to perceiving breastfeeding as emotionally beneficial. Breastfeeding was considered to strengthen emotional attachment and the mother–child bond [31]. On the contrary, those who deemed long-term breastfeeding as harmful to family relationships considered it to lead to a problem in the relationship between mother and child, and as a cause of an unhealthy attachment between mother and child. Breastfeeding was also seen as harmful to the parents’ partnership [30].

The perceptions regarding impacts on the mother’s wellbeing also varied [31]. Some health professionals found breastfeeding beyond 12 months as burdensome for the mother, which also included a view of breastfeeding as a psychologically and temporally demanding task. Breastfeeding was seen as harmful for the mother, and this view was linked to considering breastfeeding as disadvantageous and restrictive to the mother’s diet [31]. Health professionals felt that breastfeeding was physically difficult and demanding for the mother. On the other hand, perceptions regarding the impacts on the mother’s wellbeing were linked to the knowledge of health benefits for the mother. This included knowing that breastfeeding can be helpful as a birth control method and that it can also help the mother to lose weight. Health professionals also knew that breastfeeding decreases the likelihood of developing breast cancer [31].

Perceptions regarding impacts on the child’s wellbeing included knowing the health benefits for the child [31], while also a view of breastfeeding beyond 12 months as harmful to the child’s development [31] and health [30]. The health benefits of breastfeeding beyond 12 months to the child were better known than the health benefits for the mother. The unhealthy impacts on the child’s development were considered to include breastfeeding delaying the child’s development and being disadvantageous and harmful to it [30, 31].

Six studies explored the perceptions regarding appropriate breastfeeding duration [30–35]. Health professionals had varying definitions of extended breastfeeding as some defined it as up to 12 months of breastfeeding, while others perceived 24 months of breastfeeding as “long breastfeeding” [30]. Health professionals saw breastfeeding as less acceptable as the child got older [33–35]. Negative perceptions increased along with the age of the child [33, 35] and breastfeeding beyond 12 months was seen as more acceptable if the child was a younger toddler [33]. The support for breastfeeding from health professionals decreased after the first six months [34]. The variation in the views on the appropriate duration of breastfeeding was also reflected in the result that some health professionals saw breastfeeding beyond 24 months as desirable and found that children should be breastfed until over 24 months [30], and some also found it acceptable for a 3–4-year-old child to be breastfed [33]. Health professionals had varying perceptions about stopping breastfeeding [30–33]. They encouraged to stop breastfeeding more strongly as the child got older [32] and considered stopping to become more complicated when breastfeeding beyond 12 months [31]. The perceptions varied from a view that breastfeeding should be stopped before child turns six months [30] to encouraging to stop breastfeeding a 3- or 4-year-old child [32, 33].

The perceptions regarding the breastfeeding recommendations [29, 30, 34, 35] were linked to having a gap in knowledge, which had led to health professionals giving varying recommendations on the breastfeeding duration. Some health professionals had knowledge about breastfeeding duration [35] and gave recommendations about breastfeeding [29]. Despite this, there was a lack of knowledge found among health professionals related to breastfeeding and the WHO recommendations [30], and they disagreed with the recommendation to breastfeed until the child is 24 months or longer [34]. It was shown that health professionals recommended breastfeeding for up to 24 months and beyond if they had more knowledge about breastfeeding [30].

The counseling skills included providing information and warnings about breastfeeding [31, 32]. Health professionals provided clinical information about breastfeeding and its benefits and harms [31]. They considered their intentions to provide advice regarding stopping breastfeeding [32]. Health professionals also gave warnings about breastfeeding beyond 12 months [31].

### Main theme 2: Attitudes

Health professionals’ attitudes towards breastfeeding beyond 12 months were explored in four studies [30–33]. The attitudes varied and formed three themes: promotive attitudes [30–32], hostile attitudes [30–33], and passive attitudes [30, 31].

Attitudes promoting breastfeeding were reflected in the support for breastfeeding [31]. This included responding positively towards breastfeeding, approving breastfeeding, and supporting it. Promotive attitudes were also linked to positive attitudes [30, 32] and curiosity towards breastfeeding [31]. Health professionals with positive attitudes tended to find that a child should be breastfed for over 24 months. Curiosity manifested as a desire to know the reasons behind breastfeeding [31].

Hostile attitudes were noticed in four studies [30–33]. Some health professionals perceived breastfeeding questionable [30, 31]. They questioned the mother about breastfeeding and saw breastfeeding to be merely based on the mother’s own need. They also considered breastfeeding as unnecessary for the child. Hostile attitudes were linked to negative attitudes [30, 32, 33] and in some cases the health professionals’ baseline attitudes towards breastfeeding were negative. Attitudes became more negative as the child got older [32]. Some health professionals had hateful feelings towards breastfeeding [31], which came across as anger, contempt, negative emotions, and pity when seeing a breastfeeding mother. Breastfeeding was seen as awkward [31], and health professionals expressed concern towards breastfeeding and felt discomfort when seeing a breastfeeding mother. Some health professionals disparaged breastfeeding [31, 32] which manifested as a lack of respect towards breastfeeding, a feeling of discomfort towards breastfeeding [31] and negative responses towards breastfeeding [31, 32].

Passive attitudes were visible in two studies [30, 31]. Health professionals perceived their role as passive [31]. They were deferring breastfeeding and the mother’s decisions about breastfeeding. Some health professionals demonstrated indifference and saw breastfeeding as “none of my business”. They also perceived themselves as outsiders. Health professionals also had neutral attitudes [30, 31] towards breastfeeding and expressed neutral responses towards it.

## Discussion

Seven studies were reviewed in this systematic review of health professionals’ competencies regarding breastfeeding beyond 12 months. The competencies referred to health professionals' knowledge, skills, and attitudes, and all these dimensions could be discovered in the data.

The review revealed a wide range of dimensions regarding health professionals’ competencies, and it also indicated that the level of knowledge, skills and attitudes varied substantially between the professionals. Health professionals’ knowledge and skills regarding breastfeeding beyond 12 months manifested as varying perceptions about the nutritional and economic value of breastfeeding, its impact on family interactions and the wellbeing of the mother and child, breastfeeding recommendations and an appropriate duration of breastfeeding [29–35]. The counseling skills also varied [31, 32]. Health professionals considered breastfeeding beneficial and worthwhile [31], but on the other hand it was also seen as harmful and demanding, and some health professionals lacked knowledge about the WHO recommendations [30, 31]. Professionals could have knowledge of the health benefits for a child, but by contrast, some also viewed breastfeeding as harmful for the child’s health or development [30, 31]. The perceptions about the nutritional value of breastfeeding beyond 12 months varied from considering it to provide good nutrition to a view of breastfeeding causing a nutritional deficiency for the child [31]. The economic value of breastfeeding beyond 12 months was explored in one study [31] and was the only theme that did not include any contrary opinions, as health professionals saw breastfeeding as economically beneficial even when it continued beyond infancy.

Health professionals’ perceptions about the recommendations and suitable duration of breastfeeding were the most examined subject in the data. The support for breastfeeding decreased after 6 months and breastfeeding was seen as less acceptable as the child got older [33, 34]. Health professionals’ perceptions of an ideal age for stopping breastfeeding varied substantially, and this can lead health professionals to believe that breastfeeding has ceased at a certain point. This is supported by previous findings that health professionals no longer discuss breastfeeding with the mother after the child’s first year of life [4, 7]. After the child turns one, mothers may feel pressure from health professionals to cease breastfeeding without evidence-based knowledge [4, 5, 7, 16]. This is worrying, as mothers feel pressure to cease breastfeeding from society in general [38] and receive less support from others as the breastfeeding continues beyond 12 months [4]. Social stigmatization increases as the breastfed child gets older [6], and here the health professionals would play an important role in reducing the stigma and supporting the family.

The predominant, partly implicit pattern found across the data was concerned with attitudes towards breastfeeding beyond 12 months. While the health professionals had been asked about their attitudes directly [30, 31, 33] these were also manifested through the participants’ perceptions regarding an appropriate age for stopping breastfeeding or breastfeeding recommendations in each study [29–35]. Previous research has shown that health professionals support mothers breastfeeding an infant [39], but as the breastfeeding continues beyond 12 months, it is poorly understood by health professionals and the society in general [40]. The older the breastfed child, the more mothers need to justify their breastfeeding [41]. This was also visible in the data. Though there were supportive and promoting attitudes towards breastfeeding beyond 12 months [30, 31], the negative attitudes found were very strong and even hostile [30, 31, 33]. Some health professionals saw breastfeeding beyond 12 months as awkward, expressed concern about it and felt discomfort when seeing a breastfeeding mother [31]. This phenomenon can also be seen reflected in the experiences of mothers that breastfeeding beyond 12 months is not supported by health professionals [6, 10] and that health professionals’ attitudes towards breastfeeding are rude or negative [4, 41]. Mothers feel that health professionals are dismissive of breastfeeding beyond 12 months and that this dismissive attitude is based on misinformation [7]. This causes a conflict both to the mothers’ desire for evidence-based, sensitive, and individualized breastfeeding counseling [16] and the professionals’ ethical obligation to provide it [42].

The variation in the professionals’ competencies is a worrying phenomenon that is supported by previous results based on which mothers perceive the support they receive is dependent on professionals’ individual opinions [7] rather than evidence-based standards. This variation has led mothers to question the instructions given by health professionals [4, 7, 11], and to seek support and information for breastfeeding from peer support groups rather than healthcare providers [4, 10]. Mothers may stop consulting health professionals regarding breastfeeding beyond 12 months due to dismissive attitudes or an assumed lack of knowledge [4, 6, 7, 9, 11]. This may reduce the families’ confidence in professionals and hinder families’ willingness to seek help from professionals even when needed. A lack of support from health professionals not only causes dissatisfaction and mistrust [9] but may also interfere with medical care if health professionals do not take breastfeeding into account [10].

Breastfeeding education related to breastfeeding beyond 12 months is insufficient worldwide [12], which can be linked to health professionals’ lack of knowledge and negative attitudes. The positive perceptions regarding nutritional value and the impacts on the mother’s health and wellbeing were linked to knowledge [31], and health professionals recommended breastfeeding up to 24 months and beyond more often if they were more well-informed about breastfeeding [30]. The results speak in favor of increasing health professionals’ education in relation to breastfeeding beyond 12 months, and this is also a wish previously expressed by mothers [4]. It is important to note that the mothers’ and families’ needs for support related to breastfeeding beyond 12 months are different compared to breastfeeding at earlier months, and the content of breastfeeding counseling should be customized for mothers’ breastfeeding a toddler or a young child. [8] For example, a mother’s return to work can be a challenging situation that needs to be supported [10, 13].

### Limitations

There has been little research on breastfeeding beyond 12 months [43], and research on the phenomenon is important. Due to this shortage of research on the topic, a clear limitation of the present review was the limited number of studies, and due to this, saturation did not occur in all parts of the data. Two studies [30, 31] were more prominently represented in the results than the remaining five studies, and three of the themes in the Knowledge Combined with Skills main theme were formed based on only one study [31]. Nevertheless, attitudes and beliefs related to breastfeeding duration and recommendations were visible throughout the data.

The available studies presented somewhat heterogeneous aims and had been implemented using different methods. In addition, four of the studies [30–33] focused only on breastfeeding beyond 12 months. The other three studies [29, 34, 35] focused on breastfeeding during infancy, and breastfeeding beyond 12 months was only examined from the point of view of health professionals’ perceptions about breastfeeding recommendations or the duration of breastfeeding. Due to this, the contribution of each study to the themes varies.

The emphasis on attitudes and beliefs challenged the analysis. Especially the different dimensions of clinical knowledge and skills often had to be carefully identified from the descriptions of misconceptions or lack of knowledge. Because the level of knowledge varied a lot and attitudes influenced knowledge, the themes were referred to as “perceptions”, which describes the phenomenon quite well.

## Conclusions

The studies examined in the present review revealed a wide range of dimensions regarding health professionals’ competencies. It’s obvious that the level of knowledge and skills varies substantially, and attitudes influence professionals’ advice regarding breastfeeding beyond 12 months. Better knowledge is linked to more positive attitudes related to breastfeeding beyond 12 months, which emphasizes the importance of education. The results of this review can be seen as a directive when developing education for health professionals. The data synthesis process was challenged by the different tools and perspectives used in measuring the competencies of healthcare professionals related to breastfeeding beyond 12 months, which suggests that there is a need for an established and validated research tool in the future.

### Supplementary Information

**Additional file 1.** Databases and search terms.

## Data Availability

All data generated or analyzed during this study are included in this published article.

## Abbreviations

OECD: The Organisation for Economic Co-operation and Development
PRISMA: Preferred Reporting Items for Systematic Reviews and meta-analysis
WHO: World Health Organization
