# Supplementary material for: Health professionals’ competencies regarding breastfeeding beyond 12 months: a systematic review
Source: Int Breastfeed J. 2023 Oct 31;18:55. doi: 10.1186/s13006-023-00591-z (PMC10617142; doi:10.1186/s13006-023-00591-z)
Supplement: Supplementary file 1 — Additional file 1. Databases and search terms. [file 13006_2023_591_MOESM1_ESM.docx]

| **Additional file 1** | | |
| --- | --- | --- |
| **Database** | **Search terms and structure** | |
| CINAHL | Key words | AB (nurs* OR "health professional" OR "health worker" OR "health person" OR "medical staff" OR "health provider" OR midwif* OR midfiv* OR doctor* OR phycisian*)  AND  AB (knowledge OR education OR understanding OR awareness OR competence OR attitude* OR perception* OR opinion* OR thought* OR feeling* OR belief* OR view*)  AND  AB (breastfeed* OR breast feed* OR breastfed OR lactation)  AND  (beyond OR prolonged OR extended OR long-term) |
|  | MeSH terms | (MH "Breast Feeding")  AND   (MH "Professional Competence") OR (MH "Nursing Skills") OR (MH "Clinical Competence") OR    (MH "Knowledge") OR (MH "Health Knowledge") OR (MH "Nursing Knowledge") OR (MH "Professional Knowledge") OR (MH "Attitude of Health Personnel") OR (MH "Midwife Attitudes") OR (MH "Nurse Attitudes") OR (MH "Physician Attitudes") OR (MH "Attitude to Breast Feeding")  AND  (beyond OR prolonged OR extended OR long-term) |
| MEDLINE | Key words | AB (nurs* OR "health professional" OR "health worker" OR "health person" OR "medical staff" OR "health provider" OR midwif* OR midfiv* OR doctor* OR phycisian*)  AND  AB (knowledge OR education OR understanding OR awareness OR competence OR attitude* OR perception* OR opinion* OR thought* OR feeling* OR belief* OR view*)  AND  AB (breastfeed* OR breast feed* OR breastfed OR lactation)  AND  (beyond OR prolonged OR extended OR long-term) |
|  | MeSH terms | (MH "Breast Feeding")  AND  (MH "Professional Competence") OR (MH "Knowledge") OR (MH "Health Knowledge, Attitudes, Practice") OR (MH "Attitude") OR (MH ”Attitude of Health Personnel”) OR (MH ”Clinical Competence”)  AND  (beyond OR prolonged OR extended OR long-term) |
| PsycINFO | Key words | AB (nurs* OR "health professional" OR "health worker" OR "health person" OR "medical staff" OR "health provider" OR midwif* OR midfiv* OR doctor* OR phycisian*)  AND  AB (knowledge OR education OR understanding OR awareness OR competence OR attitude* OR perception* OR opinion* OR thought* OR feeling* OR belief* OR view*)  AND  AB (breastfeed* OR breast feed* OR breastfed OR lactation)  AND  (beyond OR prolonged OR extended OR long-term) |
|  | MeSH terms | (MH "Breast Feeding") OR (MH ”Lactation”)  AND   (MH "Professional Competence") OR (MH "Knowledge") OR (MH "Health Knowledge ") OR (MH "Attitudes") OR (MH ”Health Personnel attitudes”) OR (MH ”Competence”)  AND  (beyond OR prolonged OR extended OR long-term) |
| Psychology database | Key words | AB (nurs* OR "health professional" OR "health worker" OR "health person" OR "medical staff" OR "health provider" OR midwif* OR midfiv* OR doctor* OR phycisian*)  AND  AB (knowledge OR education OR understanding OR awareness OR competence OR attitude* OR perception* OR opinion* OR thought* OR feeling* OR belief* OR view*)  AND  AB (breastfeed* OR breast feed* OR breastfed OR lactation)  AND  (beyond OR prolonged OR extended OR long-term) |
|  | MeSH terms | mainsubject(breast feeding)  AND  (mainsubject(knowledge) OR mainsubject(Attitude of Health Personnel)  AND  (beyond OR prolonged OR extended OR long-term) |
| SocINDEX | Key words | AB (nurs* OR "health professional" OR "health worker" OR "health person" OR "medical staff" OR "health provider" OR midwif* OR midfiv* OR doctor* OR phycisian*)  AND  AB (knowledge OR education OR understanding OR awareness OR competence OR attitude* OR perception* OR opinion* OR thought* OR feeling* OR belief* OR view*)  AND  AB (breastfeed* OR breast feed* OR breastfed OR lactation)  AND  (beyond OR prolonged OR extended OR long-term) |
| Scopus | Key words | AB (nurs* OR "health professional" OR "health worker" OR "health person" OR "medical staff" OR "health provider" OR midwif* OR midfiv* OR doctor* OR phycisian*)  AND  AB (knowledge OR education OR understanding OR awareness OR competence OR attitude* OR perception* OR opinion* OR thought* OR feeling* OR belief* OR view*)  AND  AB (breastfeed* OR breast feed* OR breastfed OR lactation)  AND  AB (beyond OR prolonged OR extended OR long-term) |
| Cochrane Library | Key words | AB (nurs* OR "health professional" OR "health worker" OR "health person" OR "medical staff" OR "health provider" OR midwif* OR midfiv* OR doctor* OR phycisian*)  AND  AB (knowledge OR education OR understanding OR awareness OR competence OR attitude* OR perception* OR opinion* OR thought* OR feeling* OR belief* OR view*)  AND  AB (breastfeed* OR breast feed* OR breastfed OR lactation)  AND  (beyond OR prolonged OR extended OR long-term) |
|  | MeSH terms | (MH "Breast Feeding")  AND  (MH "Knowledge") OR (MH "Attitude of Health Personnel") |
